# Portable wireless and fibreless fNIRS headband compares favorably to a stationary headcap-based system

**DOI:** 10.1371/journal.pone.0269654

**Published:** 2022-07-14

**Authors:** Christopher L. Friesen, Michael Lawrence, Tony G. J. Ingram, Megan M. Smith, Eric A. Hamilton, Christopher W. Holland, Heather F. Neyedli, Shaun G. Boe

**Affiliations:** 1 Laboratory for Brain Recovery and Function, Dalhousie University, Halifax, Nova Scotia, Canada; 2 Axem Neurotechnology, Halifax, Nova Scotia, Canada; 3 Department of Psychology and Neuroscience, Dalhousie University, Halifax Nova Scotia, Canada; 4 Cognitive and Motor Performance Laboratory, Dalhousie University, Halifax, Nova Scotia, Canada; 5 School of Health and Human Performance, Dalhousie University, Halifax, Nova Scotia, Canada; 6 School of Physiotherapy, Dalhousie University, Halifax, Nova Scotia, Canada; Tokai University, JAPAN

## Abstract

This study’s purpose is to characterize the performance of a prototype functional near-infrared spectroscopy (fNIRS) headband meant to enable quick and easy measurements from the sensorimotor cortices. The fact that fNIRS is well-suited to ergonomic designs (i.e., their ability to be made wireless, their relative robustness to movement artifacts among other characteristics) has resulted in many recent examples of novel ergonomic fNIRS systems; however, the optical nature of fNIRS measurement presents an inherent challenge to measurement at areas of the brain underlying haired parts of the head. It is for this reason that the majority of ergonomic fNIRS systems that have been developed to date target the prefrontal cortex. In the present study we compared the performance of a novel, portable fNIRS headband compared with a stationary full headcap fNIRS system to measure sensorimotor activity during simple upper- and lower-extremity tasks, in healthy individuals >50 years of age. Both fNIRS systems demonstrated the expected pattern of hemodynamic activity in both upper- and lower-extremity tasks, and a comparison of the contrast-to-noise ratio between the two systems suggests the prototype fNIRS headband is non-inferior to a full head cap fNIRS system regarding the ability to detect a physiological response at the sensorimotor cortex during these tasks. These results suggest the use of a wireless and fibreless fNIRS design is feasible for measurement at the sensorimotor cortex.

## Introduction

Functional near-infrared spectroscopy (fNIRS) is gaining popularity as a modality of functional neuroimaging. While less temporally precise than methods measuring the electromagnetic properties associated with neural activity, and less spatially precise than functional magnetic resonance imaging (fMRI), fNIRS is more spatially precise than most electromagnetic methods, and more temporally precise than fMRI (not being limited by the need to serially measure slices of the brain). Moreover, recent innovations in light-emitting diode (LED), silicon photo diode (SiPD), and lithium battery technologies have together opened up the possibility space for fNIRS systems to be built in ergonomic ways that enable new research, clinical, or consumer brain-computer-interface (BCI) applications. All this positions fNIRS well to contribute to the goal of using functional neuroimaging to provide value in contexts outside the laboratory (the so-called ‘neuroergonomics’ movement [1, 2]).

However, while there have been many published articles demonstrating the ability to easily take fNIRS measurements from the prefrontal cortex in a naturalistic setting [3–6], as well as the ability to take measurements in a naturalistic setting using whole headcap-based systems [7, 8], one aspect holding back fNIRS in this regard is the fact that it is much more difficult to take measurements from parts of the head that tend to be covered by hair; this is reflected in the fact that no fNIRS system that measures from haired regions of the head enables independent device set-up by the individual donning the device. This limitation precludes fNIRS from applications where the ability to take hemodynamic measurements quickly from sensorimotor, parietal, and/or occipital regions are required—for example, in applications relating to physical rehabilitation following brain injury, motor learning for skill acquisition, or the use of steady state visual stimulation for diagnostic purposes in concussion.

When taking measurements from areas other than the prefrontal cortex, an fNIRS system must (1) have its light-transmitting parts (usually a light guide of some type) in a properly oriented position tangential and sufficiently proximate to the scalp (ideally abutting it); (2) ensure the interface between its light-transmitting-parts and the scalp are such that a sufficient amount of light is not occluded and/or absorbed by hair; and (3) to accomplish this across a variety of head shapes and sizes, (4) while remaining comfortable. For full head cap fNIRS devices, the headcap’s elasticity serves as a robust solution to the first and third challenges; the ability to allow the experimenter to quickly attach and detach optodes (attached by fiber optic cable) from their location on the headcap, then manually dislodge and comb hair away from the area underneath the optodes’ intended position, has emerged as a simple and effective solution to the second problem; while varying sizes of elastic headcaps, coupled with the ability to set the pressure with which a cap’s individual optodes press against on the scalp (using springs), allows these systems to optimize the trade-off between optical coupling and comfort for all measurement locations, across a range of varying head shapes and sizes.

Unfortunately, the use of these solutions (headcaps and experimenter intervention) precludes these fNIRS systems from being used independently (i.e., to be set up by the individual donning the device) and/or quickly (i.e., enabling a <1 minute set up time). Thus, there is a need to develop fNIRS systems that can take valid measurements through hair that do not use these solutions optimized for experimenter intervention.

The present study tested a prototype fNIRS system meant to measure from the sensorimotor cortex (SMC) that is wireless, fibreless, completely head-mounted, and does not employ a headcap to take measurements. This fNIRS prototype is a preliminary iteration of a device meant to enable (among other things) independent, at-home sensorimotor BCI applications, such as at-home neurofeedback during stroke rehabilitation [9].

This study tested the ability of this prototype fNIRS system to measure a physiological signal at SMC during a simple unilateral upper-extremity and bilateral lower-extremity movement task in healthy participants >50 years of age. Individuals >50 years of age were selected to age-match this sample to patients in need of post-stroke rehabilitation, potential future users of a neuroergonomic system. Moreover, in a within-subjects design we also collected data on these tasks with an established headcap-based fNIRS system. The data collected from these two systems were then compared by assessing their conformity to the expected pattern of SMC lateralization (with contra-lateralized increases in brain activity expected in the unilateral upper-extremity tasks, and more medially located increases in brain activity expected for the bilateral lower-extremity task), as well as a quantitative comparison of each system’s contrast-to-noise ratio (CNR).

## Materials and methods

### Participants

Twenty healthy participants over the age of 50 (M = 61.1; SD = 10.1; 10 female, 19 right-handed as per the Edinburgh handedness inventory [10]) who were not experiencing any physical disability, and had no history of neurological disease or insult were recruited to participate in a single-session study which took place at, and was approved by the ethics board of, Dalhousie University. Recruitment of older individuals was chosen to be representative of the end user of the fNIRS device being developed (i.e., stroke survivors).

### fNIRS devices and sensor configuration

**Fig 1:**
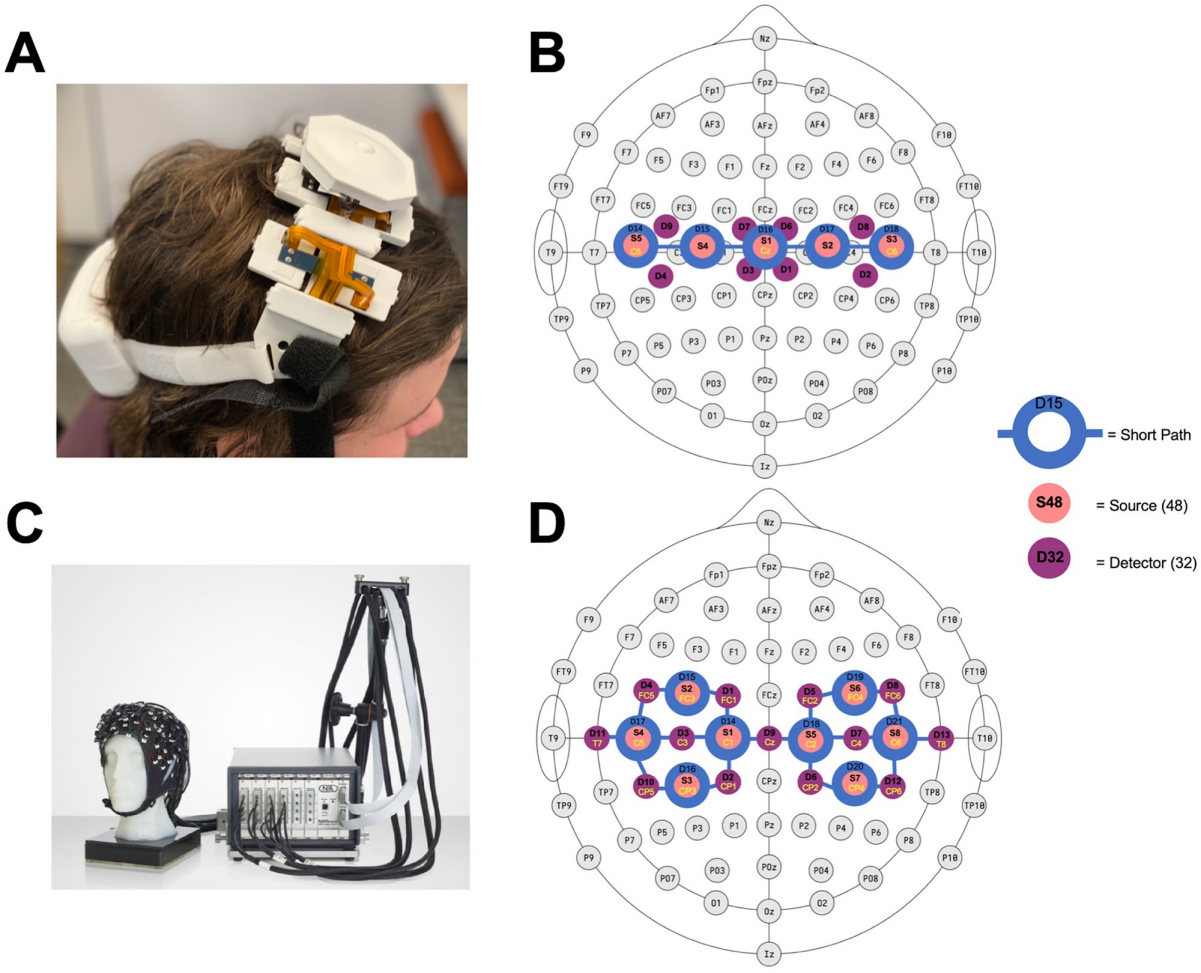
fNIRS systems used in present study. A: prototype fNIRS device. B: Array of optical components included in the fNIRS prototype. The central three detectors (being 3cm from 4 long-path LEDs) enabled 4 measurement locations each, with the two detectors on either end (being 3cm from 2 long-path LEDs) enabling two, resulting in a total of 16 measurement locations. C: NIRScout device. D: the NIRScout’s array of optical components supporting 28 measurement locations.

The fNIRS prototype used in the present study (which had been used in one prior, preliminary feasibility study [11]) was powered by a lithium battery attached to a headband of optical components; the device utilized Bluetooth low energy and supports an 8 x 2 grid of 16 unique cerebral hemodynamic measurement locations (see Fig 1A). The device is meant to be worn at the apex of the head (i.e., approximately where over-the-ear headphones sit) to enable measurement over the brain’s sensorimotor region bilaterally. Given the preliminary nature of this study, we chose to utilize this fNIRS prototype in two locations: with the device’s center detector positioned at Cz (according to the International 10–20 System), as well as 1cm anterior to this location. Given the two rows of 8 measurement locations are separated by 2cm, this allows for analysis to be conducted on a continuous grid of 4 x 8 locations.

The prototype fNIRS device contained both long-path (3cm from the detector; 745 and 850nm), as well as short-path (8mm from the detector; 735 and 850nm) channels. The collection of short-path readings has been shown to improve fNIRS measurement of cerebral hemodynamics by allowing for the removal of information reflecting hemodynamic activity superficial to the brain (i.e., within the scalp) [12]. The long-path LEDs were attached to the headband by individually articulating springs (Fig 1A; see S1 File for more information), allowing the device to adjust to the shape of users’ heads in the sagittal plane, while the use of a flexible central band (which contained the SiPDs and short-path LEDs) allowed for adjustment in the coronal plane. Importantly, all optical components (i.e., LEDs and SiPDs) were butt coupled to light pipes which enabled light transmission to and from the scalp. Compared with fiber optic cable, these light pipes were both broader and lower durometer; their relatively broad, concave tip was designed to enable them to perform their function even while standing atop some hair follicles (given the presence of a larger aperture compared with emission by fiber optic cable; see S1 File for more information); whereas their relatively low durometer allowed them to be combed through hair by simply shuffling the device back and forth on the head (a procedure that can be done by the individual wearing the device), whilst both remaining comfortable and not breaking. This combination of parts that are fixed in place and one-size-fits-all, yet flexible, and “softer” light transmitting parts than is typically utilized in fNIRS systems, serve to solve the challenges to gaining fNIRS measurements through hair (discussed in the Introduction) in a way that may enable measurements to be taken without experimenter intervention.

The NIRScout system contains 8 LED sources and 16 avalanche photodiodes which can be placed in customized configurations to measure from different regions of the brain, including the ability to take short-path measurements at all detector locations. In the present study the ‘motor montage’ specified by the manufacturer was used (see Fig 1D), as this montage includes 28 measurements across the same sensorimotor areas the fNIRS prototype’s measurement grid spanned.

### Experimental procedure

In a single experimental session, participants completed a demographic questionnaire, the Edinburgh handedness inventory, and three simple motor tasks: (1) left- and (2) right-handed ball-squeezing tasks, and (3) a seated marching task. Participants completed 10 trials of each task, completing a total of 30 trials in a randomized order. Each trial consisted of a 10 second active period and a 30 second rest period. During all tasks, participants were seated in a chair with their fingers affixed into the finger holes of a pliable, foam-like exercise ball. Auditory and visual cues instructed them to either squeeze the left or right ball at approximately 1 Hz, or to raise and lower their heels off the ground at approximately the speed of a typical walking pattern, alternating their left and right heels. For the ball-squeezing task they were instructed to use moderate force—enough so that the ball deforms, but not so much that they experience muscle strain or fatigue. Given the purpose of this study was to compare the results yielded from two fNIRS systems, force levels were not normalized to participants’ maximum voluntary contraction; rather, the use of the same exercise ball, with the same instructions, together with counterbalancing the order in which fNIRS systems were used to obtain data (see below), were done to match force level between conditions. Given a requirement of an ergonomic fNIRS system to be comfortable for the user, participants were asked to quantify, on a 10-point Likert scale [13] the level of discomfort/pain felt while wearing the prototype fNIRS system (approximately one hour across both measurement locations the fNIRS prototype was used at). The scale was anchored by the descriptors ‘No pain/discomfort at all’ (1) and ‘Almost too painful to continue’ (10). The continuum of responses also included the descriptors ‘moderately uncomfortable’ (3), ‘quite uncomfortable and/or slightly painful’ (5), and ‘moderately painful’ (8).

Participants completed these blocks of 30 trials under three conditions where the fNIRS measurements being taken differed: two while fNIRS measurements were taken with a prototype fNIRS device (at two measurement locations) and one while measurements were taken with the NIRScout fNIRS system [14–16]. While both measurement conditions utilizing the prototype fNIRS device were completed sequentially, the order of which system was used first was counterbalanced across participants. The order of the two measurement conditions using the prototype fNIRS system was also counterbalanced.

### fNIRS acquisition and pre-processing

Both devices had short-path emitters co-located with all detectors, thus acquiring one short-path measurement for every long-path measurement taken. The system-wide sample rate was 5.4 Hz for the fNIRS prototype, and 7.8 Hz for the NIRScout.

The Temporal Derivative Distribution Repair [17] (TDDR) algorithm for removal of motion artifacts was applied to all signals. Where the TDDR algorithm has been observed to perform best when high-frequency instrument noise is first removed, we applied a 2Hz low-pass filter to all data before performing TDDR (n.b. while TDDR as traditionally implemented has as a final step whereby the >2Hz high-frequency signal, that was initially filtered-out, is added back in, given this >2Hz signal represents noise in this application, this re-addition was omitted). Data were then bandpassed to the cardiac pulse band (0.5Hz to 1.5Hz), and delays in the manifestation of the pulse were computed across paths (arbitrarily selecting a reference path, which was treated as having no delay). This procedure was repeated (starting with the TDDR output each time) to measure delays in the bands associated with respiration (0.15Hz to 0.30Hz) and Meyer waves (0.05Hz to 0.15Hz). Only the data from the typically-lower-noise 850nm signal on a given path was used to calculate these delays, as it can be assumed that while delays vary from path to path, they should be the same for each wavelength on a given path.

Next the data were converted from received light levels to relative concentrations of oxyhemoglobin (ΔHbO) and deoxyhemoglobin (ΔHb) using the modified Beer Lambert equations [18] (with the mean of the 10s preceding task onset used as the reference necessitated by these equations). Data from the short-paths (for ΔHbO and ΔHb separately) was then submitted to a structural equation model (SEM) to estimate latent common influences (note that while Canonical Correlation, CC, has been used for a similar purpose [19], it has be shown that CC is a special case of SEM [20], where SEM permits more realistic/useful specification of error residuals at the measurement-level than the latent level)). This was performed four times, first on the unfiltered ΔHbO and ΔHb, then on the same data after filtering to each of the three ranges of expected frequency content (cardiac pulse, respiration, Meyer waves); for the data in each of these three distinct, physiological-signal-approximating bands, the previously estimated delays were removed by interpolation prior to estimation of the latent common signal. The resulting four latent common signals were then regressed out of all long-path and short-path ΔHbO and ΔHb channels (for each, adjusting the latents for their respective delay), thereby removing influence of these common signals of any magnitude.

Having removed common latent influences from all channels, we then (again, for ΔHbO and ΔHb separately) filter out remaining physiological noise from the short-path data (reflecting topographically-varying physiological residuals). All short-path channels were again filtered to the frequency bands of expected noise signals (cardiac pulse, respiration, Meyer waves), whereupon the previously estimated delays were again removed by linear interpolation; these delay-corrected short-path signals, together with the unfiltered short-path data, were then regressed out of the data from their associated long-path channel.

Each long-path channel was then bandpassed to the expected frequency range of the BOLD response (0.01Hz to 0.1Hz) and Correlation-Based Signal Improvement (CBSI) [21] was used to further enhance the CNR of the ΔHbO data. While the application of CBSI enables improvements in CNR by exploiting the inverse relationship between ΔHbO and ΔHb, it should be noted that because of the statistical dependency it creates between the ΔHbO and ΔHb data, only ΔHbO will be utilized in the final analysis (with HbO chosen in lieu of Hb due to its higher reliability [22]). After this the CBSI-corrected ΔHbO was again bandpassed to the BOLD frequency range, and finally baselined to the mean of the 5s period preceding the onset of the task.

It should be noted that prior to filtering throughout these procedures, the signal was padded at both the beginning and end with a time-reversed copy of itself that was in turn linearly attenuated to zero at the new beginning and end. A first order Butterworth filter was then applied to both the padded signal and a time-reversed copy of the padded signal, and after un-reversing the output of the latter application, the two outputs were averaged and padding removed.

### Bayesian modelling of ΔHbO data

Hierarchical Bayesian modelling was used to examine the distribution of ΔHbO across participants during each motor task. Specifically, the 10s task period was modelled as a linear slope (given the expectation of a monotonic increase in ΔHbO during the task period) during this period on each trial. The model nested measurement location, fNIRS system and task independently within a 3-level hierarchy, in which the mean slope for a given subject (μ_subject_) was a random normal deviate from a group mean slope (μ_group_): μ_subject_ ~ normal(μ_group_, σ_subjs_), the slope for a given trial (μ_trial_) was a random normal deviate from the participant’s mean slope: μ_trial_ ~ normal(μ_subject_, σ_trials_); and finally, the observations through time on a given trial were random normal deviates from that expected given the trial slope: obs ~ normal(time × μ_trial_, σ_obs_). Weakly-informed priors for all parameters were employed such that the gross variability observed in the data (quantified by computing the standard deviation across all datapoints, SD_obs_) informed on the general scale of the parameters. Specifically, μ_group_ ~ normal(0, SD_obs_), σ_subjs_ ~ weibull(2, SD_obs_), σ_trials_ ~ weibull(2, SD_obs_), σ_obs_ ~ weibull(2, SD_obs_). The cmdstan MCMC sampler [23] was used to generate posterior samples reflecting the posterior probability distributions on the model parameters (no samples encountered divergent transitions; all chains showed exhibited convergence (rhat<1.01) for all parameters; and no parameters exhibited low effective sample size for tail quantities).

### CNR calculation

The measurement performance of the fNIRS prototype system and NIRScout system were compared via their respective CNRs, [24] which used the slope during the task period as the numerator and σ_trials_ (variability in slope across trials) as the denominator. Moreover, given the expectation of a spatially specific response across each fNIRS system’s measurement array, and that the exact topography of this response might differ between participants, for each participant only the location (in each task and system) with the maximum median posterior slope was used. Then, for each sample in the posterior from that location, that participant’s slope was divided by that sample’s value for σ_trials_, yielding a value for CNR, for each sample in the posterior for that participant. These participant CNRs were then collapsed to a mean in each sample in the posterior for each task and system.

## Results

### Comfort questionnaire results

Overall, participants indicated a low level of discomfort with the prototype fNIRS device, evidenced by an average response of 2.07 (SD– 1.01). This value indicates responses fell squarely between ‘No pain/discomfort at all’ (1) and ‘moderately uncomfortable’ (3).

### fNIRS analysis & results

**Fig 2:**
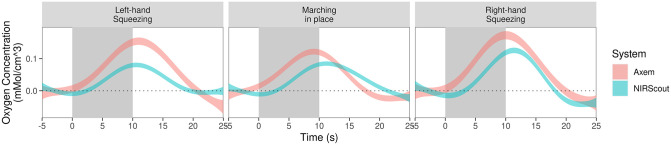
ΔHbO time series at most active measurement location during each motor task in each fNIRS system. For each motor task, the ΔHbO timeseries (95% confidence ribbons) from the measurement location which had the largest increase in ΔHbO for each fNIRS system are plotted; grey band mark the task period, while the dotted black line marks the mean of the 10 second period preceding the task. See S1 Fig for a plot depicting the ΔHbO timeseries data from all locations from both fNIRS systems.

After pre-processing, the data consisted of multiple trials of timeseries for each task condition at each location for each system and each participant. To visualize a representation related to an average timeseries across participants in each task, location and system, we first collapsed across trials (within each task, location, system and participant) to a mean timeseries using a Generalized Additive Model (GAM), where GAMs are a powerful tool to characterize timeseries data in a manner that flexibly accommodates non-linearity in a data-driven manner. Specifically, we fit the GAMs by generalized cross-validation, yielding a single timeseries per fit (i.e., for each task, location, system and participant), whereupon GAM is again employed to obtain a timeseries that reflects a mean across participants, and specifically the 95% confidence ribbons of a mean time course across participants (see Fig 2 and S1 Fig). For all three tasks and both fNIRS systems, the expected increase in ΔHbO at the onset of the task period [25] was observed: i.e., a lateralized ΔHbO increase in both unilateral hand squeezing tasks (towards the hemisphere contralateral to the hand being used), and an increase at medial locations in the marching task. These distributions of ΔHbO values conform to our neuroanatomically informed priors—of contra-lateralized data in the unilateral upper-extremity tasks, and bilateral lower-extremity tasks.

**Fig 3:**
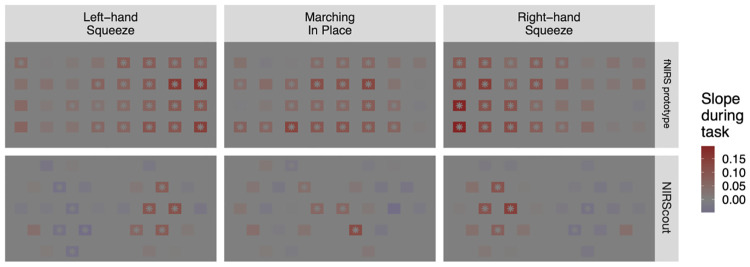
Mean ΔHbO during each motor task for each fNIRS system. Topographic maps of group mean slope (in units of mMol/mm^3^) during task. Colors indicate posterior median; locations with posterior distributions in which zero falls outside the 95% credible interval are marked with an asterisk.

Posterior samples for the group mean slope (as described in 2.5) were also obtained for each task (Fig 3; also see S1 Table for a listing of all values). Consistent with the previous figure, we see the expected contralateral activation during the squeezing tasks and medial activation during the marching task, again with rather more spatial selectivity in the NIRScout system compared to the fNIRS prototype system.

**Fig 4:**
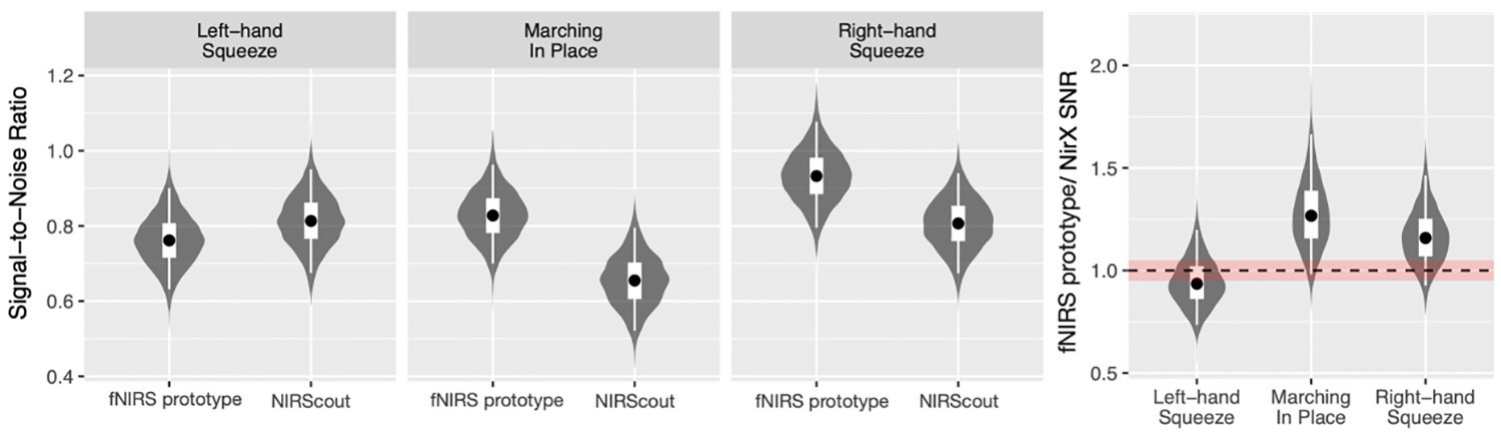
Posterior distributions of CNR ratios between fNIRS systems. Posterior distributions for CNR in each task for each fNIRS system as well as the CNR difference ratio between fNIRS systems (right-most pane)—i.e., the CNR of an fNIRS prototype system divided by the CNR of the NIRScout system (with 1 meaning no differences). Grey violins depict the mirrored density-smoothed distributions, black dots depict the posterior median, thick white rectangles depict the 50% credible interval and thin white lines depict the 95% credible interval. Red bands in the ratio plot depict the range of ratios from 0.95 to 1.05.

These posterior values were then used (as described in 2.6) to generate CNR values (reflecting the size of the measured response proportionate to the variability of that response across trials for each fNIRS system and task (collapsed across participants; see S1 Table for a listing of all values). For these values a difference ratio was calculated between the CNR values for each fNIRS system within each motor task (see Fig 4, right-most figure), by dividing the CNR of the fNIRS prototype system from that of the NIRScout system; a value of 1 therefore indicated no difference between the two CNR values. The 95% credible interval for the CNR difference ratios between fNIRS systems for all tasks were found to include a ratio of one, suggesting no evidence of differences between the fNIRS systems’ CNR values in either upper- or lower-extremity tasks.

## Discussion

The present study tested the validity of data collected at the SMC during unilateral upper- and bilateral lower-extremity movement from a prototype fNIRS system; it also compared these data to that of an established fNIRS system. The prototype fNIRS system had been designed to enable measurements from sensorimotor regions to be taken independently, employing a novel wire- and fibreless design; thus the present study compares the validity of fNIRS data from such a device with an fNIRS system which utilizes the traditional full headcap form factor. Healthy adults >50 years of age were selected as participants in order to be representative of stroke survivors, given that the final version of this device endeavors to provide at-home sensorimotor BCI feedback during physical rehabilitation. The fact that both fNIRS systems used in this study showed the expected pattern of increased ΔHbO values across the SMC in all three tasks (i.e., contra-lateralized increases in the unilateral upper-extremity tasks, primarily medially located increases in the bilateral lower-extremity task) indicate that data from both fNIRS systems are valid (see Fig 2). Moreover, the 95% credible interval of the ratio comparing CNR values from these two fNIRS systems included a ratio of one (i.e., no difference) for all three motor tasks (see Fig 4); therefore, these data do not permit us to make any claims about the superiority or inferiority of one system compared with another. And finally, results from the participant self-rating about the presence of any discomfort or pain while utilizing the fNIRS prototype indicated this fNIRS prototype is feasible for long periods of continuous use. While preliminary, together these results suggest the novel fNIRS prototype tested herein provides equivalent measurements of activity from the SMC during simple upper- and lower-extremity movements compared with an established fNIRS system.

However, there were notable differences in the spatial specificity of the increases in ΔHbO observed between these two fNIRS systems. In the upper-extremity tasks, the prototype fNIRS system had a consistent ΔHbO increase (i.e., such that the 95% credible interval for the ΔHbO slope did not include zero, as denoted by the presence of an asterisk at that location in Fig 3) across all 20 contra-lateral measurement locations, as well as 5 (in the left-handed task) and 2 (in the right-handed task) at ipsilateral locations; while the NIRScout system only showed this consistent increase in ΔHbO in 4 (right-handed movement) and 5 (left-handed movement) of a total 14 possible contra-lateral measurement locations, with none in the ipsilateral hemisphere. Likewise, in the lower-extremity task, the fNIRS prototype showed a consistent increase at 20 of 40 possible measurement locations, with the NIRScout system showing this consistent ΔHbO increase in just 4 measurement locations. While both fNIRS systems do show responses congruent with our neuroanatomical priors for these tasks (with the largest increases in ΔHbO at the most contra-lateral locations for the upper-extremity task, and at medial locations for the lower-extremity task), these findings suggest the NIRScout system may have greater spatial specificity compared with the prototype fNIRS system. This may be a result of the NIRScout system’s greater reliance on detectors (as opposed to emitters) within its array of optical components; given that this results in a larger ratio of short-path channels to long-path channels, it is possible the pre-processing techniques employed in the present study were more effective in removing extra-cerebral noise from the NIRScout compared with the fNIRS prototype data, though more work needs to be done to confirm this hypothesis.

These findings are notable given the differences between these two fNIRS systems in their inherent design: while the NIRScout employs a traditional headcap system, interfacing with a bundle of fiber optic cables to optimize easy set-up by an experimenter, the fNIRS prototype tested in this study employs a wireless, fibreless design, with light transmitting parts that allow for manipulation by the individual on whom measurements are being taken. While other fNIRS systems have been developed to measure at haired locations on the head without the use of fiber optic cable, these fNIRS systems either still utilize either a headcap form factor [26] or employ the use of light transmission pipes made of high durometer material (e.g., glass [27]), making them of limited use in translation to an fNIRS system meant for independent use. Also of note are the recent developments in small and wearable fNIRS systems which use silicon photomultipliers [28] or avalanche photo diodes [29] as detectors to obtain fibreless measurements at haired locations on the head; while impressive, the fact that these fNIRS systems rely on being tethered to a large unit to provide control and power to its optical components means they are not feasible for applications requiring independent use. Thus, the findings that fNIRS data from each of the systems used in this study provide equivalent cerebral hemodynamic measurements of both upper- and lower-extremity movement represents an encouraging step for future development of ergonomic, user-centric fNIRS systems that are built to measure from parts of the head that underly hair.

However, there are several important limitations to this study. Most importantly, while the fNIRS prototype tested in the present study was designed to enable wearers of the device to receive sensorimotor BCI feedback independent of a second individual assisting with device set-up, the ability for wearers of the device to set it up independently and take valid measurements was not tested. As this study involved the fNIRS prototype being set up by the experimenter in a similar manner as the NIRScout device, the results only provide a validation of this fNIRS prototype’s ability to collect valid data from the SMC; it does not provide validation of its ability to allow the wearer of the fNIRS device to take valid fNIRS measurements independently. With that said, the present study provides preliminary support for a ‘valid range’ of measurement locations within which this fNIRS prototype is capable of taking valid SMC measurements (i.e., ranging from one cm posterior to CZ to 2cm anterior to CZ), given that within each sagittal row of measurement locations there were locations (primarily at the lateral measurement locations, as illustrated at Figs 2 and 3) where an increase in oxyhemoglobin was found. Moreover, while the present study sought to investigate the feasibility of the use of the fNIRS prototype used herein, the choice to only ask participants to rate the comfort of the fNIRS prototype limits the interpretability and generalizability of these results. And finally, the study’s small sample size (short of the intended 30 participants due to COVID-19), and the choice to only include participants >50 years old limits the generalizability of these results; in particular given that taking fNIRS measurements through hair can be assumed to be easier (since follicular density is a major factor in the ability to get good fNIRS measurements on haired parts of the head [30], and this value negatively correlates with age [31]) in this population.

In conclusion, while preliminary, these data provide an encouraging indication this fNIRS prototype is indeed capable of taking valid fNIRS measurements during both upper- and lower-extremity movement, to a comparable degree as an established headcap and fiber optic cable based fNIRS system. Given that this ergonomic fNIRS prototype’s design may be further adapted to allow for independent fNIRS measurements to be taken, this study represents a transitionary but important step towards the development of a device capable of enabling users to independently take sensorimotor fNIRS measurements.

## Supporting information

S1 File Additional information on light transmission in novel fNIRS system. (DOCX)

S1 Fig ΔHbO time series during each motor task in each fNIRS system. ΔHbO timeseries (95% confidence ribbons) plotted at all measurement locations within each system (facet rows) and for each task (facet columns). Plots at each location are scaled to have common axes, the grey band marks the task period, and the black line marks the mean of the 5 seconds preceding the task.
(TIFF)

S1 Table Tabulated mean/standard error values (across participants) for all combination of measurement location, task, and fNIRS system. (DOCX)
