# Supplementary material for: Portable wireless and fibreless fNIRS headband compares favorably to a stationary headcap-based system
Source: PLoS One. 2022 Jul 14;17(7):e0269654. doi: 10.1371/journal.pone.0269654 (PMC9282617; doi:10.1371/journal.pone.0269654)
Supplement: S1 File — (DOCX) [file pone.0269654.s001.docx]

**Supplement 1. Additional Information on Light Transmission in a Novel fNIRS System**

While optimal from the perspective of light transmittance, the use of very hard material (i.e., with a Shore A durometer >90) imposes inherent design limitations on an fNIRS system designed to break new ground in the arenas of ergonomics and human factors. The other side of this trade-off is that the use of material that is *too* soft will not allow for reliable and effective light transmittance (since it will become highly deformed against the head in unpredictable ways, causing light to scatter unpredictably). The novel fNIRS device discussed in this work took a middle ground approach, using light pipes with a Shore A durometer of 70 (a value considered to be at the intersection of ‘medium soft’ and ‘medium hard’; or approximately the hardness of a tire tread); specifically the material Lumisil was used, given its transparency to wave lengths in the infrared and near-infrared range. And finally, the choice of materials was made in tandem with the design of the light pipes themselves—in this case a pipe approximately ~1cm long was chosen—again short enough to avoid excessive deformation, but long enough to provide some transmission through dense hair. And finally, of course the shape of the pipe must consider the way in which it is mated to an optical component; given that butt coupling was used in this fNIRS system, the pipe’s shape was such that the base would cover the flat plane of the surface-mounted LED, with a tapered narrowing enabling the pipe to better bypass dense hair (see Figure S1-1, left pane).


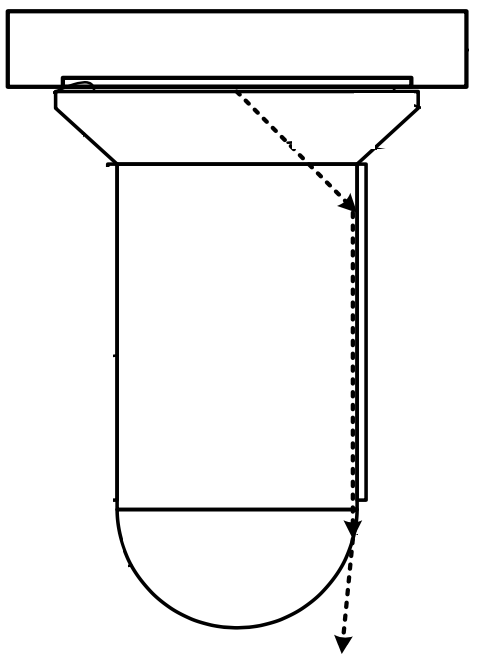
 *
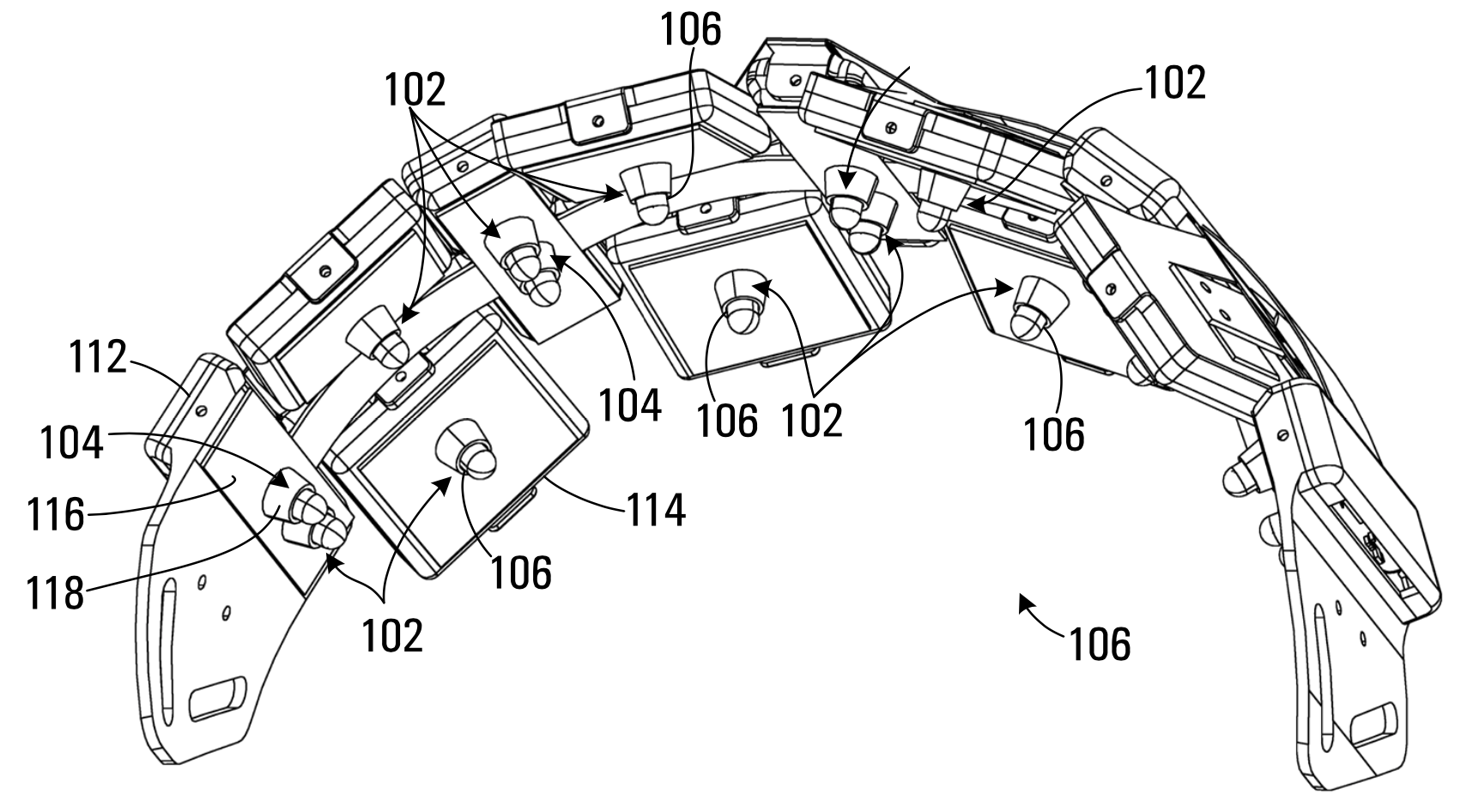
*

Figure S1-1. Left pane - Illustration of the revised light guide design implemented in fNIRS prototype. Right pane – illustration of these light guides as butt coupled to both the individually articulating long-channel LED pods (see items labeled 106) as well as the SiPDs (e.g., see item labelled 104) and short-channel LEDs (e.g., see item labelled 104).

What the use of soft light pipes in an fNIRS system means from a human factors design perspective, is that an fNIRS system can ‘automate’ the process of urging light pipes up against the head using “one-size-fits-all” spring-like mechanisms. That is, in lieu of manually adjusting the tension of springs across all optical components (a method common to fNIRS headcap designs, and indeed utilized in the NIRScout), having softer light pipes allows an fNIRS system’s optical components to be urged towards the head using springs that do not require manual manipulation; tensing all optical components identically in this way is only possible with fNIRS systems which use softer pipes, given the discomfort associated with pipes of acrylic or glass butting up against the scalp with some pressure. As discussed in the main text, in this case, all long-path LEDs were mounted on either side of a leaf springs in a pre-tensed position (see Figure S1-1, right pane), permitting these long-path LEDs to flex more-or-less in response to larger or smaller heads.
