# Supplementary figures and images for: Portable wireless and fibreless fNIRS headband compares favorably to a stationary headcap-based system
Source: PLoS One. 2022 Jul 14;17(7):e0269654. doi: 10.1371/journal.pone.0269654 (PMC9282617; doi:10.1371/journal.pone.0269654)

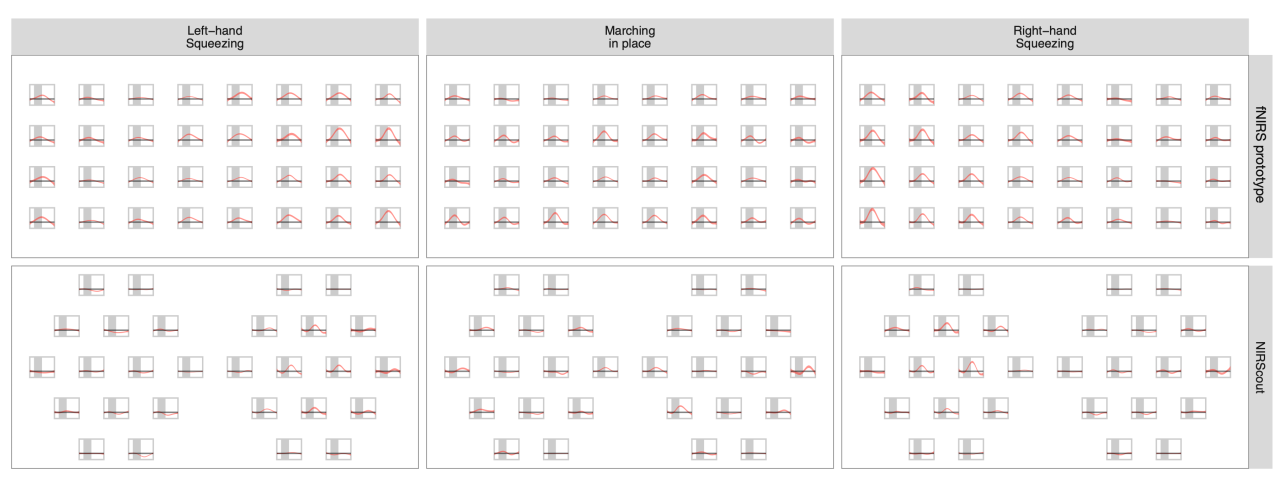

Supplement: S1 Fig — ΔHbO timeseries (95% confidence ribbons) plotted at all measurement locations within each system (facet rows) and for each task (facet columns). Plots at each location are scaled to have common axes, the grey band marks the task period, and the black line marks the mean of the 5 seconds preceding the task. (TIFF) [file pone.0269654.s002.tiff]
