# Supplementary material for: Portable wireless and fibreless fNIRS headband compares favorably to a stationary headcap-based system
Source: PLoS One. 2022 Jul 14;17(7):e0269654. doi: 10.1371/journal.pone.0269654 (PMC9282617; doi:10.1371/journal.pone.0269654)
Supplement: S1 Table — (DOCX) [file pone.0269654.s003.docx]

Supplementary Table 1a. Mean (± standard error) for the prototype fNIRS device for combinations of measurement location, task, and fNIRS system.

| Location | March | | Right Hand Squeeze | | Left Hand Squeeze | |
| --- | --- | --- | --- | --- | --- | --- |
|  | Slope | CNR | Slope | CNR | Slope | CNR |
| F1_D0_A | 39 ± 16 | 0.27 ± 0.10 | 72 ± 18 | 0.43 ± 0.09 | 24 ± 13 | 0.21 ± 0.11 |
| F1_D0_P | 100 ± 28 | 0.51 ± 0.12 | 98 ± 26 | 0.57 ± 0.12 | 62 ± 21 | 0.27 ± 0.10 |
| F1_D1_A | 2 ± 15 | 0.07 ± 0.11 | 46 ± 16 | 0.31 ± 0.09 | 12 ± 7 | 0.14 ± 0.07 |
| F1_D1_P | 43 ± 17 | 0.36 ± 0.12 | 69 ± 26 | 0.54 ± 0.14 | 19 ± 14 | 0.14 ± 0.09 |
| F2_D0_A | 44 ± 14 | 0.32 ± 0.12 | 48 ± 14 | 0.48 ± 0.10 | 77 ± 31 | 0.42 ± 0.10 |
| F2_D0_P | 88 ± 13 | 0.57 ± 0.10 | 50 ± 28 | 0.32 ± 0.15 | 64 ± 23 | 0.47 ± 0.13 |
| F2_D2_A | 45 ± 18 | 0.27 ± 0.11 | 5 ± 29 | 0.17 ± 0.12 | 72 ± 24 | 0.39 ± 0.13 |
| F2_D2_P | 106 ± 29 | 0.55 ± 0.11 | 16 ± 32 | 0.10 ± 0.14 | 73 ± 41 | 0.57 ± 0.19 |
| F3_D1_A | −15 ± 21 | −0.02 ± 0.10 | 80 ± 32 | 0.42 ± 0.10 | 4 ± 13 | 0.05 ± 0.07 |
| F3_D1_P | 33 ± 22 | 0.26 ± 0.12 | 143 ± 36 | 0.71 ± 0.13 | 20 ± 23 | 0.10 ± 0.09 |
| F3_D3_A | 25 ± 23 | 0.14 ± 0.10 | 75 ± 21 | 0.46 ± 0.10 | 42 ± 15 | 0.24 ± 0.09 |
| F3_D3_P | 32 ± 14 | 0.22 ± 0.09 | 125 ± 38 | 0.62 ± 0.14 | 32 ± 21 | 0.12 ± 0.10 |
| F4_D2_A | 27 ± 18 | 0.22 ± 0.11 | 27 ± 16 | 0.25 ± 0.10 | 71 ± 21 | 0.38 ± 0.14 |
| F4_D2_P | 16 ± 22 | 0.25 ± 0.14 | 21 ± 24 | 0.06 ± 0.13 | 145 ± 31 | 0.75 ± 0.16 |
| F4_D4_A | 20 ± 24 | 0.14 ± 0.13 | 14 ± 19 | 0.15 ± 0.08 | 63 ± 24 | 0.31 ± 0.12 |
| F4_D4_P | −6 ± 25 | 0.04 ± 0.12 | 22 ± 20 | 0.09 ± 0.12 | 158 ± 40 | 0.56 ± 0.11 |
| R1_D0_A | 53 ± 12 | 0.40 ± 0.07 | 50 ± 17 | 0.38 ± 0.13 | 26 ± 11 | 0.22 ± 0.10 |
| R1_D0_P | 86 ± 18 | 0.63 ± 0.13 | 71 ± 17 | 0.55 ± 0.13 | 53 ± 15 | 0.33 ± 0.10 |
| R1_D1_A | 39 ± 17 | 0.29 ± 0.10 | 96 ± 27 | 0.51 ± 0.11 | 34 ± 10 | 0.22 ± 0.08 |
| R1_D1_P | 119 ± 25 | 0.69 ± 0.13 | 113 ± 32 | 0.73 ± 0.17 | 19 ± 15 | 0.07 ± 0.08 |
| R2_D0_A | 48 ± 16 | 0.31 ± 0.09 | 38 ± 16 | 0.31 ± 0.10 | 47 ± 14 | 0.21 ± 0.11 |
| R2_D0_P | 90 ± 15 | 0.62 ± 0.11 | 44 ± 18 | 0.28 ± 0.11 | 54 ± 16 | 0.38 ± 0.11 |
| R2_D2_A | 81 ± 34 | 0.33 ± 0.11 | 27 ± 15 | 0.20 ± 0.10 | 65 ± 15 | 0.34 ± 0.08 |
| R2_D2_P | 80 ± 30 | 0.57 ± 0.16 | 41 ± 20 | 0.26 ± 0.12 | 88 ± 24 | 0.50 ± 0.13 |
| R3_D1_A | 10 ± 16 | 0.09 ± 0.11 | 100 ± 24 | 0.52 ± 0.10 | 11 ± 16 | 0.16 ± 0.10 |
| R3_D1_P | 46 ± 20 | 0.29 ± 0.13 | 116 ± 25 | 0.74 ± 0.14 | 19 ± 12 | 0.04 ± 0.07 |
| R3_D3_A | −9 ± 23 | 0.00 ± 0.11 | 173 ± 29 | 0.74 ± 0.12 | 50 ± 25 | 0.19 ± 0.11 |
| R3_D3_P | 52 ± 23 | 0.22 ± 0.11 | 185 ± 43 | 0.81 ± 0.24 | 57 ± 30 | 0.25 ± 0.13 |
| R4_D2_A | 35 ± 16 | 0.22 ± 0.11 | −3 ± 19 | 0.02 ± 0.09 | 83 ± 20 | 0.55 ± 0.14 |
| R4_D2_P | 55 ± 17 | 0.29 ± 0.12 | 9 ± 25 | −0.06 ± 0.16 | 88 ± 25 | 0.47 ± 0.13 |
| R4_D4_A | −11 ± 26 | −0.03 ± 0.13 | −9 ± 15 | −0.05 ± 0.10 | 83 ± 23 | 0.40 ± 0.12 |
| R4_D4_P | 4 ± 18 | −0.01 ± 0.11 | 4 ± 15 | −0.03 ± 0.10 | 141 ± 34 | 0.61 ± 0.13 |

Supplementary Table 1b. Mean (± standard error) for the NIRScout device for combinations of measurement location, task, and fNIRS system.

| Location | March | | Right Hand Squeeze | | Left Hand Squeeze | |
| --- | --- | --- | --- | --- | --- | --- |
|  | Slope | CNR | Slope | CNR | Slope | CNR |
| S1_D1 | 33 ± 17 | 0.19 ± 0.09 | 8 ± 21 | 0.19 ± 0.11 | −16 ± 12 | −0.05 ± 0.12 |
| S1_D2 | 21 ± 18 | 0.21 ± 0.12 | 26 ± 15 | 0.21 ± 0.11 | −42 ± 15 | −0.36 ± 0.10 |
| S1_D3 | −5 ± 22 | −0.08 ± 0.13 | 136 ± 27 | 0.98 ± 0.15 | −28 ± 14 | −0.36 ± 0.11 |
| S1_D9 | 50 ± 13 | 0.42 ± 0.10 | 12 ± 11 | 0.15 ± 0.11 | −8 ± 8 | 0.01 ± 0.08 |
| S2_D1 | −16 ± 10 | −0.12 ± 0.12 | −11 ± 10 | −0.11 ± 0.11 | 5 ± 12 | 0.06 ± 0.11 |
| S2_D3 | −18 ± 13 | −0.15 ± 0.09 | 95 ± 32 | 0.55 ± 0.15 | −35 ± 14 | −0.21 ± 0.09 |
| S2_D4 | 18 ± 13 | 0.21 ± 0.14 | −8 ± 10 | 0.03 ± 0.08 | −21 ± 15 | −0.06 ± 0.12 |
| S3_D10 | 7 ± 16 | 0.06 ± 0.10 | 13 ± 14 | 0.13 ± 0.10 | 5 ± 22 | 0.07 ± 0.12 |
| S3_D2 | −4 ± 18 | −0.06 ± 0.09 | 0 ± 13 | 0.07 ± 0.12 | −38 ± 12 | −0.29 ± 0.08 |
| S3_D3 | −11 ± 16 | −0.13 ± 0.09 | 65 ± 17 | 0.60 ± 0.13 | −29 ± 12 | −0.38 ± 0.13 |
| S4_D10 | 35 ± 21 | 0.23 ± 0.10 | 25 ± 17 | 0.20 ± 0.10 | 29 ± 19 | 0.28 ± 0.12 |
| S4_D11 | 22 ± 23 | 0.13 ± 0.09 | 1 ± 30 | 0.03 ± 0.13 | −17 ± 29 | 0.00 ± 0.10 |
| S4_D3 | −16 ± 18 | −0.08 ± 0.11 | 88 ± 20 | 0.66 ± 0.14 | −9 ± 17 | −0.11 ± 0.10 |
| S4_D4 | 23 ± 18 | 0.12 ± 0.08 | 30 ± 22 | 0.21 ± 0.12 | 13 ± 23 | 0.05 ± 0.11 |
| S5_D5 | 17 ± 20 | 0.04 ± 0.08 | −8 ± 8 | −0.20 ± 0.08 | 14 ± 12 | 0.25 ± 0.13 |
| S5_D6 | 100 ± 24 | 0.55 ± 0.13 | −37 ± 16 | −0.15 ± 0.10 | 48 ± 14 | 0.33 ± 0.10 |
| S5_D7 | 13 ± 14 | 0.00 ± 0.09 | −31 ± 11 | −0.30 ± 0.10 | 88 ± 17 | 0.65 ± 0.10 |
| S5_D9 | 53 ± 19 | 0.32 ± 0.08 | −12 ± 17 | −0.14 ± 0.09 | −17 ± 8 | −0.01 ± 0.09 |
| S6_D5 | −11 ± 11 | −0.18 ± 0.13 | −11 ± 7 | −0.07 ± 0.09 | −14 ± 13 | −0.15 ± 0.12 |
| S6_D7 | −21 ± 16 | −0.15 ± 0.10 | −21 ± 14 | −0.15 ± 0.10 | 80 ± 25 | 0.55 ± 0.17 |
| S6_D8 | 3 ± 18 | 0.21 ± 0.13 | 1 ± 14 | 0.02 ± 0.10 | 0 ± 22 | 0.01 ± 0.12 |
| S7_D12 | 0 ± 11 | −0.06 ± 0.08 | −1 ± 14 | −0.12 ± 0.10 | 1 ± 15 | 0.03 ± 0.14 |
| S7_D6 | 13 ± 22 | 0.00 ± 0.11 | −21 ± 12 | −0.22 ± 0.10 | 9 ± 15 | 0.12 ± 0.09 |
| S7_D7 | −21 ± 15 | −0.23 ± 0.09 | −24 ± 15 | −0.25 ± 0.11 | 75 ± 28 | 0.40 ± 0.13 |
| S8_D12 | 0 ± 23 | 0.10 ± 0.10 | 28 ± 18 | 0.06 ± 0.08 | 12 ± 24 | 0.05 ± 0.10 |
| S8_D13 | −7 ± 42 | 0.23 ± 0.14 | −17 ± 37 | −0.01 ± 0.12 | −27 ± 34 | −0.06 ± 0.11 |
| S8_D7 | −43 ± 22 | −0.25 ± 0.12 | −24 ± 22 | −0.17 ± 0.12 | 97 ± 25 | 0.54 ± 0.12 |
| S8_D8 | −23 ± 33 | 0.02 ± 0.11 | −12 ± 27 | −0.05 ± 0.13 | −7 ± 35 | 0.03 ± 0.15 |
